# Supplementary material for: Exploring the Roles of Key Mediators IKBKE and HSPA1A in Alzheimer’s Disease and Hepatocellular Carcinoma through Bioinformatics Analysis
Source: Int J Mol Sci. 2024 Jun 25;25(13):6934. doi: 10.3390/ijms25136934 (PMC11241202; doi:10.3390/ijms25136934)
Supplement: Supplementary file 1 [file ijms-25-06934-s001.zip › ijms-3021394-supplementary.pdf]

Supplementary Table S1. Information on microarray datasets obtained from Gene Expression Omnibus.

Group	GEO Data set	Platform	AD/LICH	Control
Analysis set	GSE132903	GPL10558	97	98
Validation set	GSE33000	GPL4372	310	207
Validation set	GSE36376	GPL10558	240	193
Validation set	GSE39791	GPL10558	72	72

Supplementary Table S2. GO enrichment significance of immport immune genes.

Ontology	Term	Description
BP	GO:0042221	Response to chemical
BP	GO:0048583	Regulation of response to stimulus
BP	GO:0010033	Response to organic substance
CC	GO:0005576	Extracellular region
CC	GO:0031982	Vesicle
CC	GO:0012505	Endomembrane system
MF	GO:0005102	Signaling receptor binding
MF	GO:0098772	Molecular function regulator
MF	GO:0038023	Signaling receptor activity

Supplementary Table S3. KEGG pathways of immport immune genes.

Ontology	ID	Description
KEGG	hsa05200	Pathways in cancer
KEGG	hsa04060	Cytokine-cytokine receptor interaction
KEGG	hsa05169	Epstein-Barr virus infection
KEGG	hsa05164	Influenza A
KEGG	hsa04010	MAPK signaling pathway
KEGG	hsa05168	Herpes simplex virus 1 infection
KEGG	hsa05167	Kaposi sarcoma-associated herpesvirus infection
KEGG	hsa05163	Human cytomegalovirus infection
KEGG	hsa04612	Antigen processing and presentation
KEGG	hsa04151	PI3K-Akt signaling pathway

Supplementary Table S4. GO enrichment analysis of key genes.

Ontology	Term	Description
BP	GO:0048583	regulation of response to stimulus
BP	GO:0048584	positive regulation of response to stimulus
BP	GO:0035556	intracellular signal transduction
CC	GO:0031982	vesicle
CC	GO:0005576	extracellular region
CC	GO:0005829	cytosol
MF	GO:0019899	enzyme binding
MF	GO:0005102	signaling receptor binding
MF	GO:0098772	molecular function regulator

Supplementary Table S5. KEGG pathways linked to key genes.

Ontology	ID	Description
KEGG	hsa04010	MAPK signaling pathway
KEGG	hsa05162	Measles
KEGG	hsa05160	Hepatitis C
KEGG	hsa05163	Human cytomegalovirus infection
KEGG	hsa05200	Pathways in cancer
KEGG	hsa04668	TNF signaling pathway
KEGG	hsa05161	Hepatitis B
KEGG	hsa05164	Influenza A
KEGG	hsa05169	Epstein-Barr virus infection
KEGG	hsa05165	Human papillomavirus infection

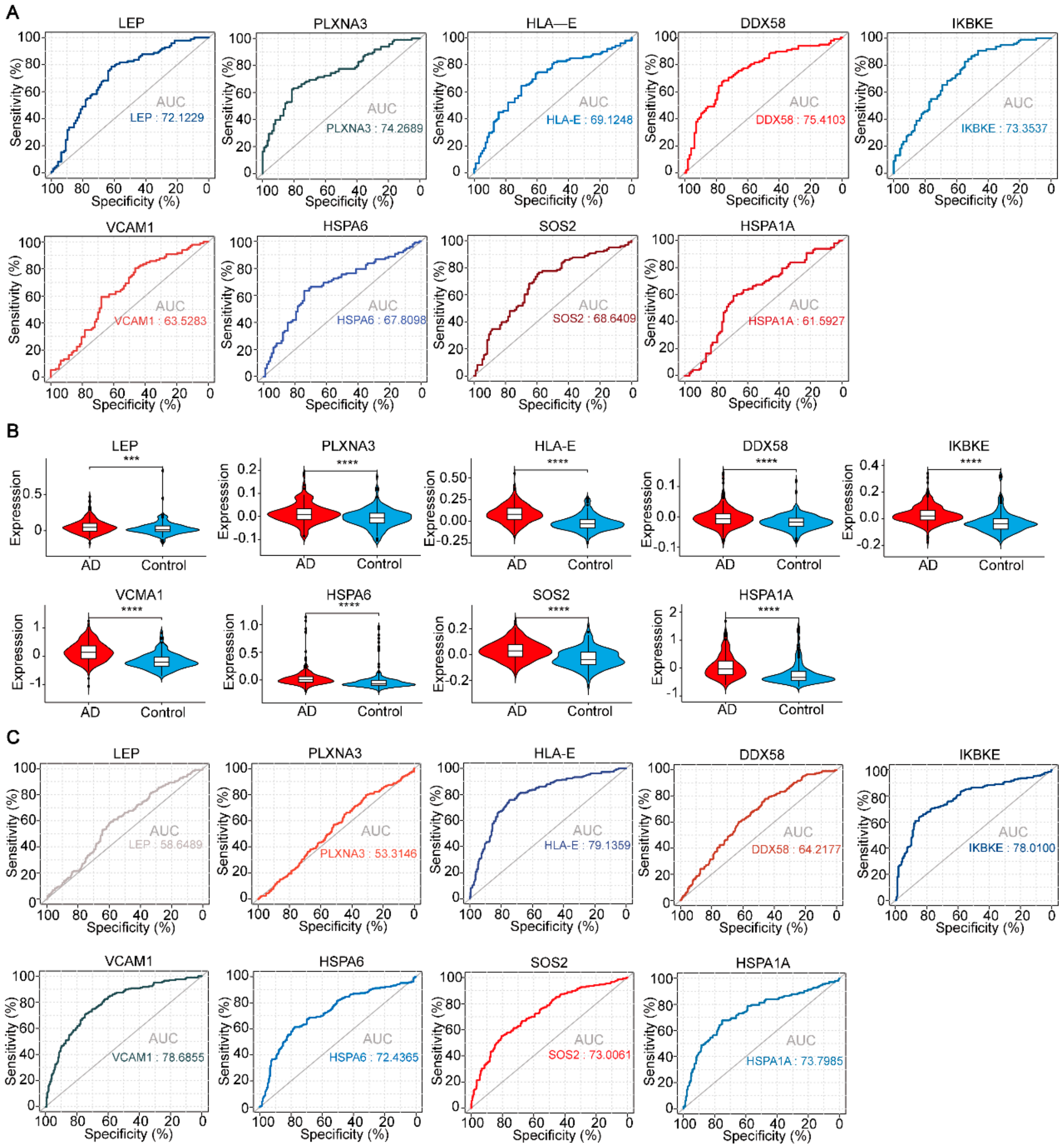

Supplementary Figure S1. Expression and Validation of Key Genes in AD. (A) ROC curves of key genes in dataset GSE132903. (B) Expression of key genes in the validation group GSE33000. (C) ROC curves of key genes in the validation group GSE33000. *** and **** indicate significant difference from the control group, *** $p < 0.001$, **** $p < 0.0001$ compared with control group.

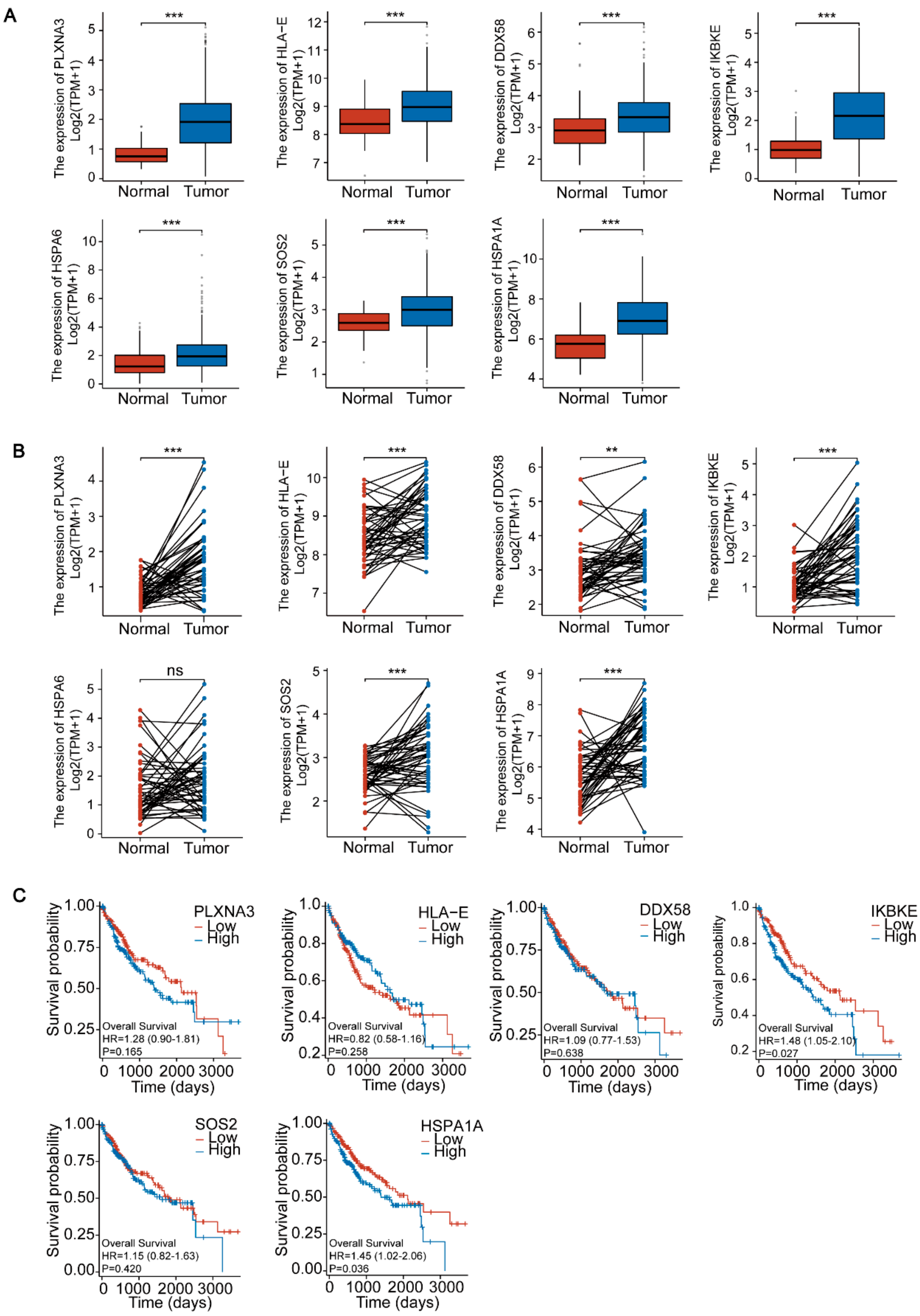

Supplementary Figure S2. Expression and Prognosis of Key Genes in LIHC. (A) Box plots illustrating the expression levels of key genes in liver samples. (B) Paired comparison plots of key genes in individual matched normal and tumor samples. (C) Kaplan-Meier survival curve analysis for key genes. ** and *** indicate significant difference from the Normal group, ** $p < 0.01$, *** $p < 0.001$ compared with Normal group.

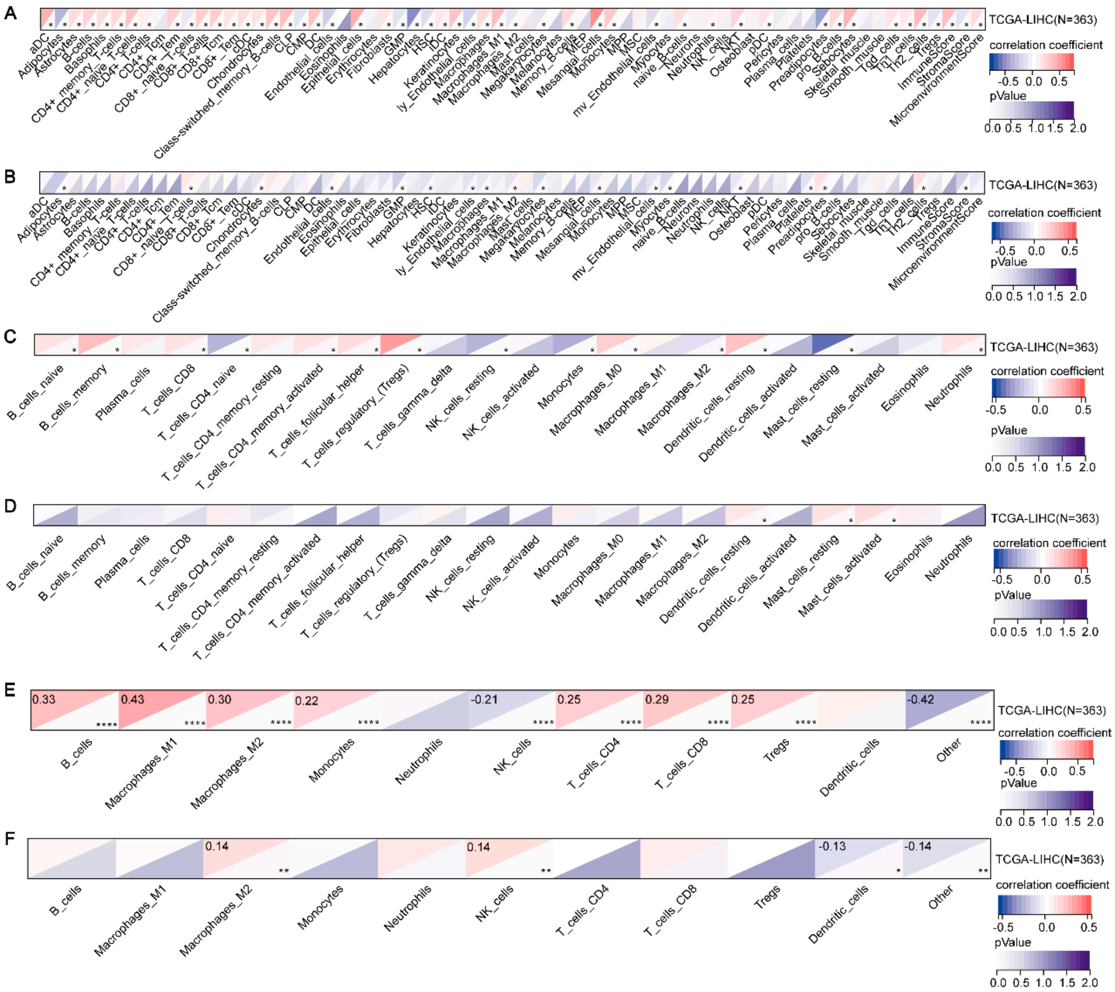

Supplementary Figure S3. Algorithmic Analysis of Immune Cell Infiltration for *IKBKE* and *HSPA1A* in LIHC. (A-B) xCELL algorithm results for *IKBKE* and *HSPA1A*. (C-D) CIBERSORT algorithm results for *IKBKE* and *HSPA1A*. (E-F) QUANTISEQ algorithm results for *IKBKE* and *HSPA1A*. *, ** and **** indicate significant difference from the control group, * $p < 0.05$, ** $p < 0.01$, **** $p < 0.0001$ compared with control group.
